# Rising Above the Limits of Critical Care ECMO: A Narrative Review

**DOI:** 10.3390/medicina61020174

**Published:** 2025-01-21

**Authors:** Pietro Bertini, Alberto Marabotti, Paolo Meani, Fabio Sangalli, Gianluca Paternoster

**Affiliations:** 1 Department of Anesthesia and Intensive Care Medicine, Casa di Cura Privata San Rossore, 56122 Pisa, Italy; 2 Intensive Care Unit and Regional, ECMO Referral Centre, Azienda Ospedaliero, Universitaria Careggi, 50134 Florence, Italy; marabottialberto@gmail.com; 3 Department of Cardiothoracic Surgery, Heart and Vascular Centre, Maastricht University Medical Centre, 6229 HX Maastricht, The Netherlands; paolo.meani@mumc.nl; 4 Department of Anaesthesia and Intensive Care, ASST Valtellina e Alto Lario, University of Milano-Bicocca, 23020 Sondrio, Italy; fabio.sangalli@unimib.it; 5 Department of Health Science, Anesthesia and ICU, School of Medicine, University of Basilicata San Carlo Hospital, 85100 Potenza, Italy; paternostergianluca@gmail.com

**Keywords:** ECMO, critical care, respiratory failure, cardiac surgery, perioperative medicine

## Abstract

Extracorporeal membrane oxygenation (ECMO), an advanced life support method, was developed to treat severe cardiac and pulmonary failure in critically ill patients. ECMO was previously used to treat ARDS, cardiogenic shock, and after heart or lung transplant. It has since become a versatile therapeutic and surgical tool. When conventional methods fail, this technique works well for high-risk procedures such as tracheal resections, ventricular tachycardia ablations, and complicated percutaneous coronary interventions. These uses demonstrate ECMO’s ability to oxygenate and stabilize the hemodynamics in challenging clinical circumstances. Clinical studies report survival rates exceeding 60% in ECMO-assisted thoracic surgeries, underscoring its efficacy in these settings. Recent advancements, such as portable ECMO systems and artificial intelligence-driven management tools, have further enhanced the safety and effectiveness of ECMO, enabling its use in diverse clinical environments. However, challenges remain, particularly in patient selection, resource allocation, and addressing ethical dilemmas. The integration of standardized protocols and technological innovations has mitigated complications such as vascular injury and infection, contributing to improved patient outcomes. This review examines ECMO applications and integration into multidisciplinary care, its configurations, and its growing role outside the intensive care unit in elective thoracic and cardiac surgery, trauma, and non-cardiac high-risk procedures.

## 1. Introduction

Extracorporeal membrane oxygenation (ECMO) is an advanced life support system for critically ill patients unresponsive to standard respiratory and cardiac treatments. Originally designed as an adjunct to cardiopulmonary bypass systems, it has transformed into an essential element of modern critical care by temporarily replacing cardiac and pulmonary functions. The swift restoration of oxygenation and perfusion it delivers usually buys time for patients to recover, undergo heart or lung transplantation, or receive other advanced treatments.

Historically, ECMO was widely utilized in neonatology to treat meconium aspiration syndrome and persistent pulmonary hypertension. Over several decades, its scope broadened to incorporate acute respiratory distress syndrome (ARDS), severe cardiogenic shock, and primary graft failure subsequent to heart or lung transplantation in the adult or pediatric population [1,2]. Portable and biocompatible ECMO systems have facilitated its implementation in diverse environments such as emergency departments, operating rooms, and prehospital scenarios enhancing its clinical applicability [3].

Epidemiological data from the Extracorporeal Life Support Organization (ELSO) Registry highlights ECMO’s global adoption and its increasing relevance in critical care. As of 2022, over 200,000 patients worldwide have received ECMO support, with more than 100,000 surviving to discharge [4]. Furthermore, during the COVID-19 pandemic, ECMO became indispensable in managing severe respiratory failure, as highlighted by us, as we demonstrated survival differences in COVID-19-related ARDS [5].

Recent improvements have expanded ECMO’s use, establishing it as a crucial adjunct in high-risk elective operations and emergency interventions, supported by data from large-scale registry analyses and clinical studies [4]. Complex thoracic and cardiac surgeries, as well as obstetric and trauma procedures, benefit from ECMO’s capacity to sustain hemodynamic stability, improve oxygenation, and diminish perioperative problems in meticulously chosen patient groups [6,7]. Advancements in engineering, biomaterials, and critical care medicine have led to innovations such as sophisticated oxygenators, centrifugal pumps, and comprehensive monitoring systems that improve the safety and efficacy of ECMO. Furthermore, integrating artificial intelligence into ECMO management could enhance real-time decision making, maximize resource use, and improve patient outcomes [8].

Although ECMO has transformative potential, it poses considerable hurdles, such as substantial resource demands, bleeding and thrombotic problems, and ethical dilemmas with end-of-life care. These concerns highlight the necessity of continuous research, consistent treatment regimens, and effective communication among multidisciplinary teams to optimize the advantages of ECMO while mitigating its risks [9].

This review offers a thorough examination of the expanding uses, fundamental mechanisms, and related problems of ECMO. This discussion analyzes ECMO’s application in elective and emergency procedures, highlighting future potential and demonstrating its capacity to address deficiencies in traditional therapies while establishing a foundation for novel strategies in critical and perioperative medicine.

Recent improvements have expanded ECMO’s use, establishing it as a crucial adjunct in high-risk elective operations and emergency interventions. Complex thoracic and cardiac surgeries, as well as obstetric and trauma procedures, benefit from ECMO’s capacity to sustain hemodynamic stability, improve oxygenation, and diminish perioperative problems in meticulously chosen patient groups [4,5]. Research from many studies substantiates ECMO’s adaptability in tackling complex clinical issues across diverse medical fields.

Advancements in engineering, biomaterials, and critical care medicine have led to innovations such as sophisticated oxygenators, centrifugal pumps, and comprehensive monitoring systems that improve the safety and efficacy of ECMO. Furthermore, integrating artificial intelligence into ECMO management could enhance real-time decision making, maximize resource use, and improve patient outcomes.

Although ECMO has transformative potential, it poses considerable hurdles, such as substantial resource demands, bleeding and thrombotic problems, and ethical dilemmas with end-of-life care. These concerns highlight the necessity of continuous research, consistent treatment regimens, and effective communication among multidisciplinary teams to optimize the advantages of ECMO while mitigating its hazards [7].

This review offers a thorough examination of the expanding uses, fundamental mechanisms, and related problems of ECMO. This discussion analyzes ECMO’s application in elective and emergency operations, highlighting future potential and demonstrating its capacity to address deficiencies in traditional therapies while establishing a foundation for novel strategies in critical and perioperative medicine.

## 2. The ECMO Circuit and Its Configurations

The ECMO system can temporarily substitute cardiac and pulmonary functions, allowing these essential organs to rest and recover. The apparatus consists of essential components that work together to provide reliable and efficient clinical support. Cannulas, specifically engineered for blood extraction and reinfusion, are large-bore catheters introduced into the vascular system to ensure optimal flow and pressure dynamics. The centrifugal pump propels blood through the system by minimizing shear forces that could damage erythrocytes, effectively functioning as an artificial heart within the extracorporeal circulation framework and ensuring reduced hemolysis. The oxygenator, often called the artificial lung, removes carbon dioxide from circulation and increases oxygen levels, thereby replicating the vital function of the respiratory system to maintain the necessary gaseous equilibrium in the blood for sustaining life. Additionally, a heat exchanger regulates blood temperature to maintain normothermia and prevent serious patient complications that could result from temperature variations in the circuit. To reduce the risk of thrombotic and inflammatory reactions, each component is interconnected via biocompatible tubing in the extracorporeal circuit.

**Figure 1:**
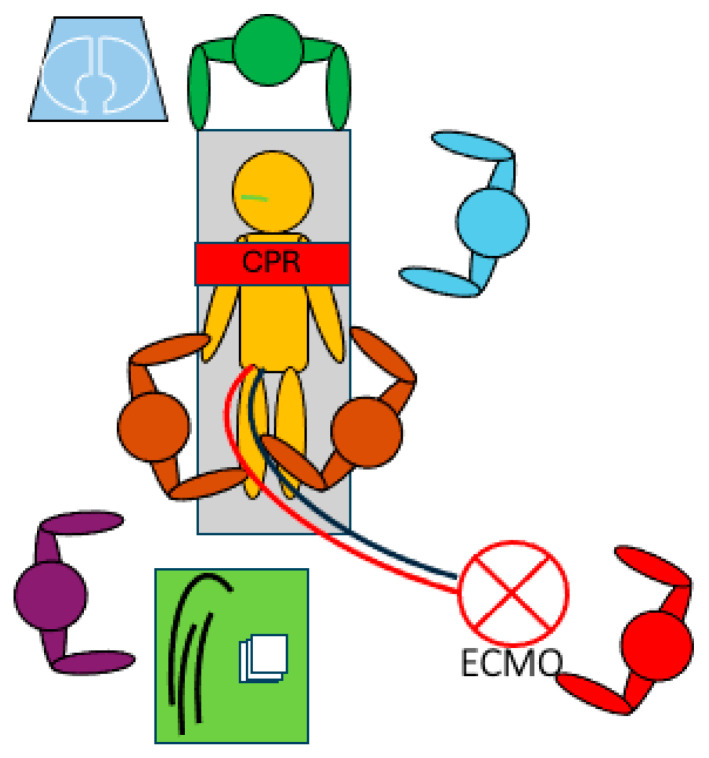
Typical ECMO cannulation sequence and scenario during ECPR.

**Figure 2:**
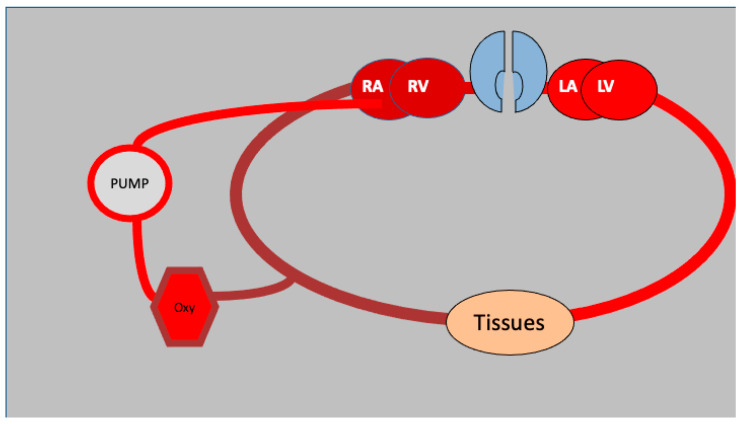
VV ECMO circuit schematics: deoxygenated blood is withdrawn from the venous circulation, oxygenated (Oxy) and pushed via the pump into the right atrium (RA). RV: right ventricle, LA: left atrium, LV: left ventricle.

**Figure 3:**
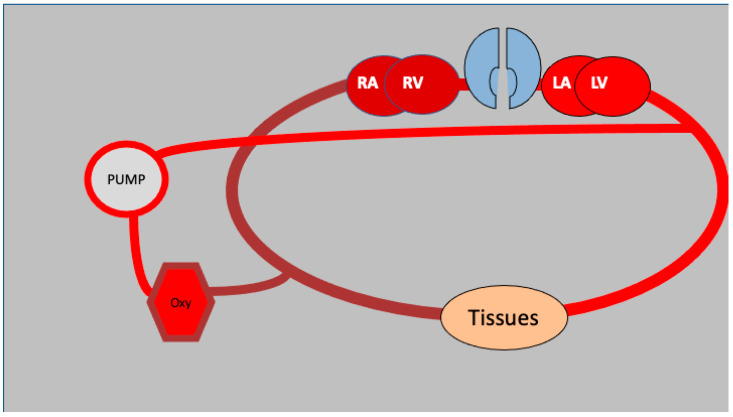
VA ECMO circuit schematics: deoxygenated blood is withdrawn from the venous circulation, oxygenated (Oxy) and pushed via the pump into the systemic circulation bypassing the native lungs and heart. RA: right atrium, RV: right ventricle, LA: left atrium, LV: left ventricle.

When selecting a suitable mode of ECMO for the patient, the clinical presentation of the patient should be taken into consideration (Figure 1). Acute respiratory distress syndrome (ARDS) and severe pneumonia are two examples of respiratory failure that are regularly seen in critical care environments [10]. Veno-venous extracorporeal membrane oxygenation (VV-ECMO) is a technique that is frequently used to treat respiratory failure for these medical conditions. VV-ECMO virtually bypasses pulmonary function during the patient’s lung recovery (Figure 2). This ensures that oxygenation and carbon dioxide removal are carried out effectively throughout the duration of the support, thus limiting any further impairment. As a result of its ability to support both cardiac and respiratory functions, veno-arterial extracorporeal membrane oxygenation (VA-ECMO) has the potential to be an acceptable treatment for cardiogenic shock as well as severe circulatory collapse or cardiac arrest [11,12]. It is possible for VA-ECMO to maintain systemic perfusion by bypassing cardiac and pulmonary function, which allows for the preservation of critical organ blood flow (Figure 3). This is possible despite the severe hemodynamic instability that is present. The utilization of this method ensures that vital organs receive an adequate amount of perfusion. In order to address difficult clinical circumstances, such as patients who require both pulmonary and myocardial recovery, hybrid techniques that incorporate parts of veno-venous (VV) and veno-arterial (VA) extracorporeal membrane oxygenation (ECMO) configurations are also being developed [13]. These hybrid strategies combine the capabilities of both systems.

## 3. Unconventional Uses of ECMO

ECMO has evolved far beyond its original role in critical care, where it was primarily used for severe cardiac and respiratory failure in intensive care units. Once considered a last resort, ECMO is now a versatile tool for life support across various clinical scenarios. Advances in technology and expanding applications have enabled its use in contexts far removed from its initial purpose. Case reports and studies highlight its effectiveness in both critical and elective situations, offering life-saving support in high-risk cases. In elective thoracic and cardiac procedures, ECMO plays a vital role. In thoracic surgeries, particularly complex tracheal resections and reconstructions, ECMO provides continuous oxygenation and ventilation when conventional methods are insufficient. This ensures a stable environment for surgeons while reducing intraoperative risks. Similarly, in high-risk cardiac interventions, such as ventricular tachycardia ablations, ECMO maintains hemodynamic stability, mitigating complications and improving outcomes. Structural cardiac procedures, including transcatheter aortic valve replacement (TAVR) and catheter-based mitral valve repair, also benefit from ECMO’s ability to sustain circulation and manage hemodynamic instability in high-risk patients.

Beyond elective procedures, ECMO has shown promise in trauma care, poisoning, peripartum cardiomyopathy, severe septic shock and massive pulmonary embolism.

### 3.1. Elective Thoracic Procedures

A recent ELSO Registry analysis reported a survival rate of 39.8% in all thoracic neoplasm and a high survival rate (73.5%) in the sub-group of upper airway neoplasm, confirming ECMO efficacy and safety [14]. The majority of patients received veno-venous ECMO (63.3%). Indeed, the primary indication of extracorporeal support seems to be the need to overcome technical issues in ventilation in patients with often reduced lung function due to thoracic neoplasm itself, baseline presence of lung diseases, and complications of neoadjuvant and adjuvant chemotherapy, such as immune checkpoint inhibitors, particularly PD-1 inhibitors, which are associated with a high incidence of pneumonitis [15]. Maintaining airway patency and ensuring sufficient oxygenation are critical issues faced during tracheal surgery, particularly when addressing lesions that limit airflow or necessitate complex surgical methods. These procedures frequently need interruptions in ventilation or provide anatomical challenges that make standard oxygenation techniques inadequate. In these situations, ECMO replaces the function of the lungs, so providing oxygenation and CO_2_ removal. This support enables surgeons to execute complex tracheal resections and reconstructions with improved precision and safety, particularly when conventional breathing methods are unsuitable due to the location or severity of the lesion. The provision of ECMO during these procedures enhances the technical effectiveness of the treatment and guarantees good patient outcomes, markedly diminishing perioperative hazards.

Likewise, ECMO has transformed endoscopic thoracic interventions, including minimally invasive airway and esophageal surgery. These operations frequently necessitate prolonged apnea or single-lung breathing, which can be difficult to administer using traditional methods, especially in high-risk patients. Extended apnea elevates the risk of hypoxemia and hemodynamic instability, presenting considerable complications for both anesthesiologists and surgeons. ECMO mitigates these risks by providing continuous oxygenation and systemic perfusion during the procedure, allowing surgeons to concentrate on the complex requirements of the surgery without the worry of oxygen deficiency. ECMO mitigates the necessity for harsh mechanical ventilation or elevated oxygen concentrations, hence diminishing the risk of ventilator-induced lung injury and oxygen toxicity. This is especially pertinent for patients with impaired lung function or those undergoing extended surgical procedures. The capacity to sustain minimal ventilatory pressures and volumes while ensuring optimum gas exchange signifies a notable progress in perioperative treatment, especially in high-risk thoracic procedures.

Furthermore, ECMO has proven its efficacy in cases of dynamic airway blockage, including tracheomalacia and airway stenosis. These disorders frequently present unforeseen complications during surgery, including episodes of severe blockage that may jeopardize ventilation. ECMO serves as a safeguard in these situations, enabling surgeons to tackle the underlying pathology without the critical need of preserving airway patency. This has broadened the range of surgically viable options, facilitating the treatment of illnesses previously considered excessively high-risk [16].

Clinical evidence underscores the increasing acceptance of ECMO in thoracic surgical procedures. An analysis of ECMO-assisted tracheal operations and minimally invasive thoracic procedures indicates elevated survival rates and a significant decrease in perioperative sequelae, including arrhythmias, hemodynamic instability, and extended hypoxemia. These results highlight the necessity of integrating ECMO into the repertoire of strategies for addressing intricate thoracic disorders.

### 3.2. High-Risk Cardiac Procedures

ECMO is essential in high-risk cardiac procedures. In cath lab procedures, such as ablations for ventricular tachycardia, veno-arterial (VA) ECMO stabilizes hemodynamics, enabling interventional cardiologists to identify and ablate arrhythmogenic foci without the risk of cardiac arrest [17]. This approach is especially beneficial for patients exhibiting significant left ventricular dysfunction or hemodynamic instability.

ECMO support has demonstrated benefits for safeguarded percutaneous coronary interventions (PCIs) in patients with difficult coronary anatomy or significant left ventricular dysfunction. ECMO enhances overall success rates and mitigates peri-procedural complications by maintaining coronary perfusion during these procedures [18]. When integrated with advanced imaging and catheterization techniques, ECMO presents novel opportunities for patient cohorts previously considered untreatable.

Moreover, ECMO is being increasingly employed in transcatheter aortic valve replacement (TAVR) and high-risk mitral valve interventions. Its ability to stabilize hemodynamics and address complications such as cardiac tamponade makes it essential for structural heart interventions [19,20]. Recent case reports demonstrate that ECMO-assisted TAVR produces outcomes similar to traditional methods while substantially reducing procedural risks in high-risk populations.

### 3.3. Non-Cardiac Elective Surgical Procedures and Emergency Interventions

In addition to cardiac and thoracic applications, ECMO has demonstrated significant efficacy in non-cardiac surgeries for high-risk patients. It can provide sufficient respiratory support during thoracic surgeries involving substantial mediastinal masses when traditional ventilation is inadequate [21]. ECMO has been utilized in abdominal surgeries, including oncologic resections, especially when substantial comorbidities increase perioperative risks [22,23,24,25]. In these situations, ECMO serves as a transitional support to conclusive surgery, diminishing both morbidity and mortality rates.

ECMO has also shown advantages in emergency obstetric situations, including peripartum cardiomyopathy and pulmonary hypertension. Its application in high-risk cesarean deliveries has resulted in enhanced maternal and neonatal outcomes [26]. These patients are especially susceptible to decompensation due to the hemodynamic alterations associated with pregnancy, such as increased cardiac output and vascular resistance. ECMO provides adequate oxygenation and perfusion during delivery, serving as a critical safeguard.

Advanced treatments such as extracorporeal life support is essential for patients experiencing refractory shock, hemorrhagic pleural effusions, or severe traumatic brain injuries [27,28]. ECMO in trauma patients has demonstrated promising outcomes, though with a difference between veno-venous and veno-arterial support. The meta-analysis of Zhang and colleagues reported a survival of 72.3% in veno-venous ECMO versus 39% in veno-arterial ECMO [29], highlighting that the main goal is respiratory and not hemodynamic support: hemorrhagic shock seems not a good ECMO indication.

The use of ECMO in trauma subjects is burdened by an incremental risk of bleeding, which can even be fatal. Literature reviews report bleeding incidence in line with other ECMO indications [30,31]. However, anticoagulation management demonstrated significant variability, from full heparin systemic anticoagulation to a heparin-minimized or all heparin-free strategy [31].

ECMO in toxicological exposure demonstrated high survival rates, both for veno-venous and for veno-arterial support. Its versatility includes the management of severe drug overdoses, offering circulatory and respiratory support during the critical phase of toxin elimination [32]. No specific contraindications are reported for this special group of subjects, although it seems reasonable to evaluate the brain injury carefully and to start an early neurological prognostication assessment in known toxin-abused (methanol, carbon monoxide, etc.) brains so as not to overtreat patients without a reasonable chance of a favorable neurological outcome [33].

The adaptability of ECMO increasingly encompasses other emergency situations. ECMO has been utilized in instances of septic shock associated with significant cardiac depression and refractory hypoxemia. Sepsis management typically depends on antibiotics and supportive care; however, using ECMO in specific instances has enhanced results by offering brief cardiac and respiratory assistance during the critical illness period [34]. Additionally, ECMO has been utilized in patients with significant pulmonary embolism leading to hemodynamic instability, serving as a temporary measure until definitive treatments like thrombolysis or surgical embolectomy can be administered [35].

## 4. Factors to Consider and Challenges to Overcome

The resource-intensive nature of ECMO poses significant challenges, particularly in resource-constrained environments. The high costs of equipment, disposables, and the need for a specialized team of doctors, nurses, and perfusionists create substantial financial and logistical barriers. These limitations often lead to inequities in access, disproportionately affecting patients in under-resourced areas. Ethical dilemmas arise when allocating ECMO resources, especially during crises like pandemics or natural disasters. Addressing these issues requires transparent regulations and multidisciplinary collaboration to ensure fairness and equality [36]. Extending ECMO use in cases with minimal chances of recovery introduces additional ethical and practical concerns. Prolonging life in such situations may impose emotional and financial burdens on patients and their families while straining healthcare resources that could benefit others with better prognoses. Implementing clear decision-making frameworks and ethical standards is essential to balance patient welfare with resource management. Given the finite availability of ECMO machines, ICU beds, and skilled personnel, hospitals face difficult decisions about prioritizing patients most likely to benefit. Ethical principles such as justice, fairness, and respect for autonomy are central to these decisions. Triage frameworks often prioritize patients with a higher likelihood of survival and a meaningful quality of life, especially during public health crises like the COVID-19 pandemic. To mitigate misunderstandings and build trust, transparent communication with patients and families is critical. Clear criteria for ECMO allocation should be shared openly to ensure decisions are perceived as fair and consistent. Addressing the challenges of ECMO utilization requires a balanced approach that maximizes patient benefit while adhering to ethical principles and managing limited resources effectively [37].

The implementation of cannulation procedures, whether central or peripheral, has specific dangers and problems. Central cannulation, typically conducted in the operating room or intensive care unit, carries possible consequences including hemorrhage, mediastinal infections, and vascular damage. Peripheral cannulation, usually performed on the femoral or jugular veins, is less invasive but poses risks of vascular perforation, extremity ischemia, and thromboembolic complications. Although progress in imaging technologies and guided cannulation procedures has considerably mitigated some hazards, the procedure still needs a high degree of competence and precise attention to detail. Stringent training and compliance with defined guidelines are crucial for reducing problems and safeguarding patient safety [38].

One of the most enduring issues in ECMO care is the intricate balance between mitigating thrombotic consequences and controlling bleeding risks. ECMO circuits intrinsically activate the coagulation cascade, elevating the danger of thrombus development inside the circuit and in the patient’s physiology. To mitigate this, anticoagulant medication, predominantly with heparin, is frequently offered. This, however, elevates the risk of hemorrhage, which may present as cerebral bleeding, gastrointestinal bleeding, or localized bleeding at cannulation sites. Achieving an optimal equilibrium between sufficient anticoagulation and hemorrhage prevention poses a significant problem for healthcare professionals. Monitoring coagulation measures, including active clotting time (ACT) and anti-factor Xa levels, is essential; nonetheless, attaining optimal anticoagulation targets frequently necessitates personalized changes according to the patient’s clinical condition [39].

Infections represent a notable problem linked to ECMO. The utilization of large-bore cannulas and extended exposure to extracorporeal circuits establishes entrance sites for infections and undermines the immune response. Bloodstream infections, pneumonia, and cannula site infections are prevalent infectious problems in ECMO patients. These infections elevate morbidity and mortality while complicating the clinical course by requiring supplementary measures, such as antibiotic medication or cannula replacement. Preventive strategies, such as rigorous compliance with aseptic protocols, regular surveillance cultures, and the prompt removal of superfluous invasive devices, are crucial for reducing the risk of infection. Notwithstanding these endeavors, infection management in ECMO continues to be a significant worry necessitating perpetual care [40].

In veno-arterial ECMO (VA-ECMO), particular issues associated with left ventricular (LV) function frequently occur, such as LV dilatation and compromised unloading. VA-ECMO offers circulatory assistance by diverting blood away from the heart and lungs; nevertheless, this alteration in blood flow may result in heightened afterload on the left ventricle. Consequently, the left ventricle may become inflated and overdistended, hindering myocardial recovery and elevating the risk of pulmonary edema and thrombus formation. Strategies for managing left ventricular unloading are essential for maximizing results in patients undergoing veno-arterial extracorporeal membrane oxygenation (VA-ECMO). Common methodologies encompass the utilization of intra-aortic balloon pumps (IABPs), percutaneous ventricular assist devices (e.g., Impella), or direct surgical venting techniques. Every method entails distinct risks and advantages, necessitating that the selection of the strategy be customized to the patient’s particular clinical situation [41].

### Success Rate, the Importance of Teamwork and the Advancement of Innovative Technologies

Because ECMO is a highly complex and resource-intensive procedure, its management necessitates a multidisciplinary approach, involving healthcare professionals with diverse expertise. The collaborative efforts of these specialists are critical to ensuring optimal care during a patient’s critical period.

Intensivists are central to the management of critically ill patients on ECMO. They oversee patient assessment, make decisions regarding the initiation and management of ECMO, and coordinate care among the various specialists involved. Cardiologists, particularly those specializing in heart failure, arrhythmias, and advanced cardiac care, play a key role in managing ECMO patients with primary cardiac conditions. Pulmonologists are also needed when ECMO is used for respiratory failure (veno-venous ECMO). They are involved in diagnosing and managing the underlying pulmonary conditions. Perfusionists are specially trained technicians responsible for managing the ECMO circuit. They ensure the proper functioning of the ECMO machine and maintain adequate blood flow during the procedure. Nurses are paramount to the daily management of ECMO patients in the intensive care unit (ICU), providing continuous monitoring and bedside care. Cardiothoracic surgeons or, in some cases, vascular surgeons are involved in the surgical aspects of ECMO placement, as well as addressing potential complications. Pharmacists play a crucial role in managing medications, particularly those related to anticoagulation and sedation, ensuring they are appropriately prescribed and monitored. Nutritionists or dietitians provide critical support by ensuring that ECMO patients receive adequate nutrition tailored to their needs. Social workers and psychologists offer emotional and psychological support for patients and their families, helping them navigate the stress and uncertainty associated with ECMO care. They assist families in making difficult decisions, including transitioning to palliative care when necessary, and address mental health concerns such as anxiety, depression, or PTSD that may arise during or after ECMO treatment especially when the patient is awake during the ECMO run [42].

Clinical ethicists or bioethicists provide guidance on the ethical dilemmas that may arise during ECMO care, especially in resource-limited settings or when conflicting interests exist between patients, families, and healthcare providers. A recent study by Eiki Nagaoka et al. analyzed the impact of a multidisciplinary approach in low-volume ECMO centers. The authors concluded that multidisciplinary decision making and teamwork significantly contributed to survival rates, highlighting the importance of this approach, particularly in resource-constrained settings [43]. A multidisciplinary approach to ECMO care is vital for delivering high-quality, patient-centered care to critically ill patients. By combining the expertise of intensivists, surgeons, nurses, perfusionists, and other specialists, healthcare teams can address the complex, life-threatening conditions that necessitate ECMO. This teamwork ensures both the technical and emotional needs of patients are met, ultimately improving outcomes and enhancing the quality of care provided.

Clinical studies and registry data indicate that ECMO is effective in improving survival rates and surgical outcomes. In elective cardiac and thoracic surgeries, survival rates often surpass 60%, accompanied by a significant reduction in intraoperative complications. Simultaneous technological advancements have significantly enhanced the safety and efficacy of ECMO. Advancements including biocompatible circuits, portable systems, and artificial intelligence (AI)-enhanced monitoring have expanded its clinical applications. Portable ECMO devices have transformed care by allowing the transport of critically ill patients and providing support in resource-constrained settings. Simultaneously, AI integration offers real-time analytics and predictive insights, enhancing patient management and reducing complications [44].

Artificial intelligence (AI) is increasingly being applied to enhance various aspects of extracorporeal membrane oxygenation (ECMO) management. One significant application is in patient selection. AI algorithms can analyze extensive datasets, including patient histories, laboratory results, and clinical parameters, to identify individuals who are most likely to benefit from ECMO therapy. Another critical area is predictive analytics. AI models can forecast potential complications or outcomes, such as risks of bleeding or thrombosis, the likelihood of successful weaning from ECMO, or overall survival probability. AI also plays a role in real-time monitoring and decision support. These systems can continuously assess key parameters such as blood flow, oxygenation, carbon dioxide removal, hemodynamic stability, and circuit pressures. AI tools can facilitate the early detection of circuit malfunctions or significant changes in a patient’s condition. By alerting clinicians to abnormalities, suggesting interventions, and reducing cognitive load, AI enhances the efficiency and accuracy of ECMO management.

Recent research highlights the transformative potential of AI in ECMO. In a study by Stephens et al., a deep neural network called the ECMO Predictive Algorithm (ECMO PAL) was evaluated on a retrospective cohort of 18,167 patients from the international Extracorporeal Life Support Organization (ELSO) registry [45]. The authors concluded that ECMO PAL is the first AI-powered survival prediction model specifically trained and validated using a large, international patient cohort. This work demonstrates how global registry data can be effectively utilized for AI-based prognostication in complex critical care settings.

AI holds great promise for revolutionizing ECMO management by improving efficiency, safety, and patient outcomes. However, its successful integration requires rigorous validation and careful implementation to ensure reliability and efficacy.

## 5. Conclusions

Although it was once only used as a measure of last resort in intensive care units, ECMO has since evolved into a comprehensive technique that has a wide range of applications. Its ability to improve results for high-risk patients is shown by the fact that it can be useful in unusual settings. In spite of this, it is essential to conduct additional research and make technological advancements in order to address the complications associated with this technique and to improve its efficiency. The utilization of this technology is expected to expand in the field of critical and perioperative medicine, which will result in a transformation of care standards across a variety of clinical specialties.

## Data Availability

No new data were created or analyzed in this study.
